# Functional protein biomarkers based on distributions of expression levels in single-cell imaging data

**DOI:** 10.1093/bioinformatics/btaf182

**Published:** 2025-04-21

**Authors:** Misung Yi, Tingting Zhan, Hallgeir Rui, Inna Chervoneva

**Affiliations:** Department of Statistics & Data Science, College of Software and Convergence, Dankook University, Suji-gu, Gyeonggi-do 16890, Korea; Division of Biostatistics & Bioinformatics, Department of Pharmacology, Physiology & Cancer Biology, Sidney Kimmel Medical College, Thomas Jefferson University, Philadelphia, PA 19107, United States; Division of Cancer Biology, Department of Pharmacology, Physiology & Cancer Biology, Sidney Kimmel Medical College, Thomas Jefferson University, Philadelphia, PA 19107, United States; Division of Biostatistics & Bioinformatics, Department of Pharmacology, Physiology & Cancer Biology, Sidney Kimmel Medical College, Thomas Jefferson University, Philadelphia, PA 19107, United States

## Abstract

**Motivation:**

The intra-tumor heterogeneity of protein expression is well recognized and may provide important information for cancer prognosis and predicting treatment responses. Analytic methods that account for spatial heterogeneity remain methodologically complex and computationally demanding for single-cell protein expression. For many functional proteins, single-cell expressions vary independently of spatial localization in a substantial proportion of the tumor tissues, and incorporation of spatial information may not affect the prognostic value of such protein biomarkers.

**Results:**

We developed a new framework for using the distributions of functional single-cell protein expression levels as cancer biomarkers. The quantile functions of single-cell expressions are used to fully capture the heterogeneity of protein expression across all cancer cells. The quantile index (QI) biomarker is defined as an integral of an unspecified function which may depend linearly or nonlinearly on a tissue-specific quantile function. Linear and nonlinear versions of QI biomarkers based on single-cell expressions of ER, Ki67, TS, and CyclinD3 were derived and evaluated as predictors of progression-free survival or high mitotic index in a large breast cancer dataset. We evaluated performance and demonstrated the advantages of nonlinear QI biomarkers through simulation studies.

**Availability and implementation:**

The associated R package Qindex is available at https://CRAN.R-project.org/package=Qindex and R package hyper.gam is available at https://github.com/tingtingzhan/hyper.gam. Examples of R code and detailed instructions could be found in vignette quantile-index-predictor (https://CRAN.R-project.org/package=hyper.gam/vignettes/applications.html#quantile-index-predictor).

## 1 Introduction

Current and emerging technologies in cancer research are geared toward capturing gene, mRNA, or protein expression in each single cell of a tumor tissue. Single-cell transcriptomics is increasingly used to elucidate the heterogeneity of gene and mRNA expressions in cancer and stroma cells and to phenotype different populations of immune cells in the stroma. Meanwhile, single-cell protein expressions have more direct relevance to disease progression, and cancer biomarkers capturing tumor heterogeneity of various protein expressions in cancer and stromal cells are needed for prognosis and classification.

Advanced quantitative pathology platforms provide tools for segmentation of multiplex immunofluorescence (mIF) immunohistochemistry (IHC) staining images and quantification of protein expressions in each cell. Single-cell protein biomarkers can be broadly classified as functional or phenotypic (Wrobel *et al.* 2023). *Phenotypic markers*, such as cluster of differentiation (CD) markers, identify different cell types, including but not limited to different populations of immune cells. *Functional markers* are quantitative in nature (the quantity matters biologically) and measure protein levels in one or more cell types, with the primary interest in cancer cells. In this work, we focus on functional protein markers.

Single-cell mIF-IHC data include *cellular signal intensity* (CSI) of each protein expression and spatial coordinates of the cell centroids. Spatial heterogeneity of protein expression may be captured using the marked point processes framework (Stoyan and Stoyan 1994). For phenotypic markers, the analysis is primarily focused on distances between cancer cells and immune cells or spatial co-localization of different types of cells (e.g. Keren *et al.* 2018, Creed *et al.* 2021, Canete *et al.* 2022, Vu *et al.* 2022, 2023, Masotti *et al.* 2023).

For functional protein markers, the *mean signal intensity* (MSI) of the protein expression, which is a simple average of CSI levels within the cancer cell compartment, remains the most commonly used approach in quantitative pathology. Recently, we proposed an approach for the analysis of functional protein biomarkers based on *quantile functions* of CSI distributions, capturing the entire distributions of single-cell protein expression but not the spatial information (Yi *et al.* 2023a). There is a limited number of methods proposed for analysis of single-cell functional protein markers taking into account spatial localization of cells (e.g. Chervoneva *et al.* 2021, Seal and Ghosh 2022, Seal *et al.* 2022). Alternatively, functional protein markers may be categorized and analyzed as multi-type marked point patterns, but it is well known that categorization of continuous predictors may lead to the loss of power or bias (Harrell 2015).

Single-cell analysis of functional protein markers is computationally intense when spatial localization of cells is taken into account, and interpretation of spatial analysis results remains challenging for mainstream use. Meanwhile, the MSI biomarkers that ignore both spatial and distributional heterogeneity provide valuable biomarkers for a wide variety of functional proteins. Therefore, it is important to evaluate the added value of taking into account distributional heterogeneity and/or spatial heterogeneity for meaningful and efficient analysis of singe-cell functional protein expression levels.

In case of independence between marks and locations in the marked point processes (a.k.a. random labeling), the spatial locations do not add any information about the distributions of marks (Baddeley *et al.* 2015). Hence, for functional protein CSIs independent of cell locations, it is sufficient to consider only the distribution of CSIs and ignore the locations of cells to capture all information about the heterogeneity of protein expression. Spatial independence between CSIs and locations of cells can be addressed by testing the hypothesis of random labeling using the global envelopes tests (Myllymäki *et al.* 2017). For single-cell mIF-IHC data, the results of the independence test tend to vary between tumor tissues even for the same protein expression. For example, for markers analyzed in Chervoneva *et al.* (2021), the null hypothesis of independence between CSI values and spatial locations of cells was rejected for 39%–45% of the tissues. For markers considered in this report, the assumption of independence was rejected for 63%–82% but was appropriate for 18%–37% of tissues. Mixed results of testing independence between CSI values and cell locations for functional mIF-IHC markers highlight the need to justify performing spatial analysis of *functional markers* because the spatial approaches available are still limited and methodologically complex, computationally intensive, and have more challenging interpretation.

In this work, we develop biomarkers that take in account between- and within-tissue variability of a functional protein’s CSIs but not spatial locations of cells. We propose a nonlinear quantile index (QI) nlQI biomarker allowing the association between the CSI quantile function and outcome to vary nonlinearly in both the functional domain and the value of the quantile function. While ignoring the spatial information, nlQI biomarker optimizes the use of CSI information. For selected functional proteins with mixed results of spatial independence testing, we compare the performance of the nlQI biomarkers to the standard MSI, the linear QI QI biomarker (Yi *et al.* 2023a), the optimal quantile (OQ) biomarkers of Yi *et al.* (2023b), and the spatial biomarkers AUcMean and AUcVar (Chervoneva *et al.* 2021). Using simulations, we demonstrate that the nlQI biomarker yields higher predictive power than the *QI* biomarker when between-tissue variability in CSI distributions is substantial.

## 2 Materials and methods

The proposed QI biomarkers capture the full heterogeneity of the protein expressions by utilizing the empirical quantile functions of CSI distributions. We assume that the CSI data are available from M independent biological samples (tissues or subjects), and for each subject i, 1≤i≤M, there are $n_{i}$ CSI observations. For a sample $S_{i}=\{x_{ij},1\leq j\leq n_{i}\}$ of CSIs from subject i and probability p, 0<p<1, the empirical quantile function $Q_{n_{i}}(p)$ is defined as the *k*th order statistic of the sample $S_{i}$, where k is such that (k−1)/n<p<k/n.

For each subject i, with the quantile function $Q_{n_{i}}(p)$ of the CSI distribution, we define the linear QI $QI_{i}$ as the integral of $Q_{n_{i}}(p)$ multiplied by a weighting function β(p),

(1) $$QI_{i}=\int_{0}^{1}\beta (p)Q_{n_{i}}(p)dp.$$

The unknown common weighting function β(p) is estimated by fitting a functional general linear model (FGLM) of James (2002) for outcomes from the exponential family of distributions or by fitting a linear functional Cox model (LFCM) (Gellar *et al.* 2015) for survival outcomes. The sign of β(p) may be adjusted to ensure that higher *QI* values correspond to higher values of median CSI (Yi *et al.* 2023a).

The nonlinear QI $\mathrm{nlQ}I_{i}$ is defined as

(2) $$\mathrm{nlQ}I_{i}=\int_{0}^{1}F(p,Q_{n_{i}}(p))dp,$$

where F(·,·) is an unspecified bivariate twice differentiable function. Thus, (2) captures nonlinear dependence of $\mathrm{nlQ}I_{i}$ on the functional predictor $Q_{n_{i}}(p)$ if F(·,·) is nonlinear in the second argument. For outcomes from the exponential family, F(·,·) is estimated by fitting a Functional Generalized Additive Model (FGAM) of McLean *et al.* (2014),

$$g\{E(Y_{i}|Q_{n_{i}})\}=\theta_{0}+\int_{0}^{1}F(p,Q_{n_{i}}(p))dp,$$

where g is a known link function, $Y_{i}$ is the outcome for subject i, and $\theta_{0}$ is the intercept. For survival outcomes, F(·,·) is estimated by fitting an Additive Functional Cox Model (AFCM) of Cui *et al.* (2021)
 

$$\log(h_{i}(t,F,Q_{n_{i}}))=\log(h_{0}(t))+\int_{0}^{1}F(p,Q_{n_{i}}(p))dp,$$

where $h_{i}(t,F,Q_{n_{i}})$ is the hazard function for subject i and $h_{0}(t)$ is a nonparametric baseline hazard function.

For estimation, F(·,·) is represented by a tensor product of univariate penalized splines (P-splines),

(3) $$F(p,q)=\sum_{j=1}^{N_{p}}\sum_{l=1}^{N_{q}}\theta_{j,l}B_{j}^{p}(p)B_{l}^{q}(q),$$

where $B_{j}^{p}$ and $B_{l}^{q}$ are univariate P-splines on the domains of p and q, respectively. The vector of unknown coefficients θ with entries $\theta_{j,l}$ in (3) is estimated by fitting an FGAM or AFCM model to a training dataset.

For each subject l in a test dataset, $\mathrm{nlQ}I_{l}$ is computed using (2) with the estimated surface $\hat{F(p,q)}$. The resulting $\mathrm{nlQ}I_{l}$ can be used as a standard predictor of relevant clinical outcomes in a test set.

We have developed an R package Qindex that implements the computation of QI’s and nlQI’s in training and test sets. FGAM or AFCM models are fitted using the gam function of the R package mgcv. The original implementation of FGAM and AFCM models includes identifiability constraint $\sum_{i=1}^{M}\int_{0}^{1}F(p,Q_{n_{i}}(p))dp=0$. Our computations of nlQI also include the adjustment of the sign of $\hat{F(p,q)}$ to ensure that higher nlQI values correspond to higher values of median CSI. The adjustment preserves the identifiability constraint and helps interpret the results for nlQI as an outcome predictor.

##  

The IHC image data that motivates this work comes from tissue microarrays (TMAs) for a large cohort of 782 stage I–III breast cancer patients treated with surgery at Thomas Jefferson University Hospital, Philadelphia, PA. None of the patients had neoadjuvant therapy before the surgery. The primary outcome of interest is progression-free survival (PFS), defined as the time from diagnosis to the evidence of local, regional, or distant recurrence. Known prognostic factors included age, race, HER2 positivity, histologic grade, node status, and tumor size (<2, 2–5, >5 cm). The clinical follow-up ranged from 2 to 238 months with a median follow-up of 114 months.

Fluorescence-based IHC was performed individually using validated antibodies for the following proteins: proliferation marker Ki-67 (Agilent/Dako; MIB-1; cat# M7240; 1:200 dilution), estrogen receptor-alpha (ER; Agilent/Dako; SP1; cat# M3634; 1; 200 dilution), CyclinD3 (Cell Signaling; cat# DCS22; 1:50 dilution), and thymidylate synthase (TS; Cell Signaling; TS106; cat# 5653; 1:100 dilution). For each protein of interest, the tumors were co-stained with pan-cytokeratin antibodies (mouse monoclonal AE1/AE3 (Agilent/Dako; cat# M3515) or rabbit polyclonal (Agilent/Dako; cat# Z0622)) and DAPI to facilitate the detection of carcinoma cells and cell nuclei, respectively. Stained tissue slides were scanned at 20× magnification using the Scanscope laser scanner (Leica/Aperio). The cell segmentation of mIF images was performed using the Definiens Tissue Studio platform (Definiens AG, Munich, Germany). For each protein, the CSI level was computed as the average pixel intensity across all pixels within each cell. The MSI was computed as the average of the CSI levels in all cancer cells in the tissue. Log-transformed CSI levels were analyzed due to the skewness of CSI distributions.

Independence of CSI values and locations of cancer cells was tested for each protein and each tissue core using the global envelopes test (Myllymäki *et al.* 2017) based on mark-weighted K function (Penttinen *et al.* 1992) in R package GET (Myllymäki and Mrkvička 2024).

For each patient, the empirical quantile function was tabulated for p=0.01,…,0.99. The patient’s cohort was randomly split into a training set with 60% of patients (469 patients with 88 recurrences) and a test set with 40% of patients (313 patients with 59 recurrences).

For each protein, AFCM and LFCM models with PFS outcome and functional predictor Q(p) were fitted to the data in the training set. To illustrate the proposed methodology with a binary endpoint, we also modeled high (score 3) versus low (score 1–2) pathologist’s mitotic index as dependent on Q(p) using the FGAM model. The R package Qindex was used to compute sign-adjusted $\hat{\beta (p)}$ and $\hat{F(p,q)}$ the training set and to compute $QI_{i}$ and $\mathrm{nlQ}I_{i}$ for every subject i in the test set.

The actual number of knots used for fitting AFCM and FGAM models was smaller than the default in gam to improve the smoothness of results and avoid overfitting. A number of proteins were considered. Examples presented include only proteins with CSI quantile functions that were significant as functional predictors in FGAM or AFCM model. The significance testing is implemented in the R package mcgv.

In the test set, nlQIs and QIs were computed using $\hat{F(p,q)}$ and $\hat{\beta (p)}$ estimated in the training set. For comparison, we also computed the standard MSI and the functional protein markers AUcMean and AUcVar (Chervoneva *et al.* 2021) based on CSIs and spatial locations of the cancer cells.

The resulting QI, nlQI, AUcMean, and AUcVar biomarkers as well as MSI levels for each protein were considered as continuous predictors of PFS in univariate and multivariable Cox proportional hazards models and as predictors of high pathologist’s mitotic index in the logistic regression models fitted to the test set. For multivariable analysis of PFS and mitotic index status, we first developed parsimonious models including only known clinicopathologic risk factors. Then, each protein biomarker was added to the parsimonious model to evaluate its prognostic value while adjusting for significant clinicopathologic risk factors. Continuous *QI* and *nlQI* biomarkers were standardized by subtracting the median and dividing by the interquartile range since the units of *QI* and *nlQI* have no biological meaning. The validity of the proportional hazard assumption was evaluated using the R package survival.

## 3 Results

### 3.1 Prediction of PFS

For the PFS outcome, ER and TS CSI quantile functions were significant as functional predictors in the AFCM model fitted in the training set. ER mediates signaling by estradiol and promotes breast cancer cell growth, while TS is an enzyme that helps create and repair DNA by converting deoxyuridine monophosphate to deoxythymidine monophosphate.

The null hypothesis of independence between CSIs and locations of cells was rejected for 82% of ER images and 64% of TS images.

**Figure 1.**
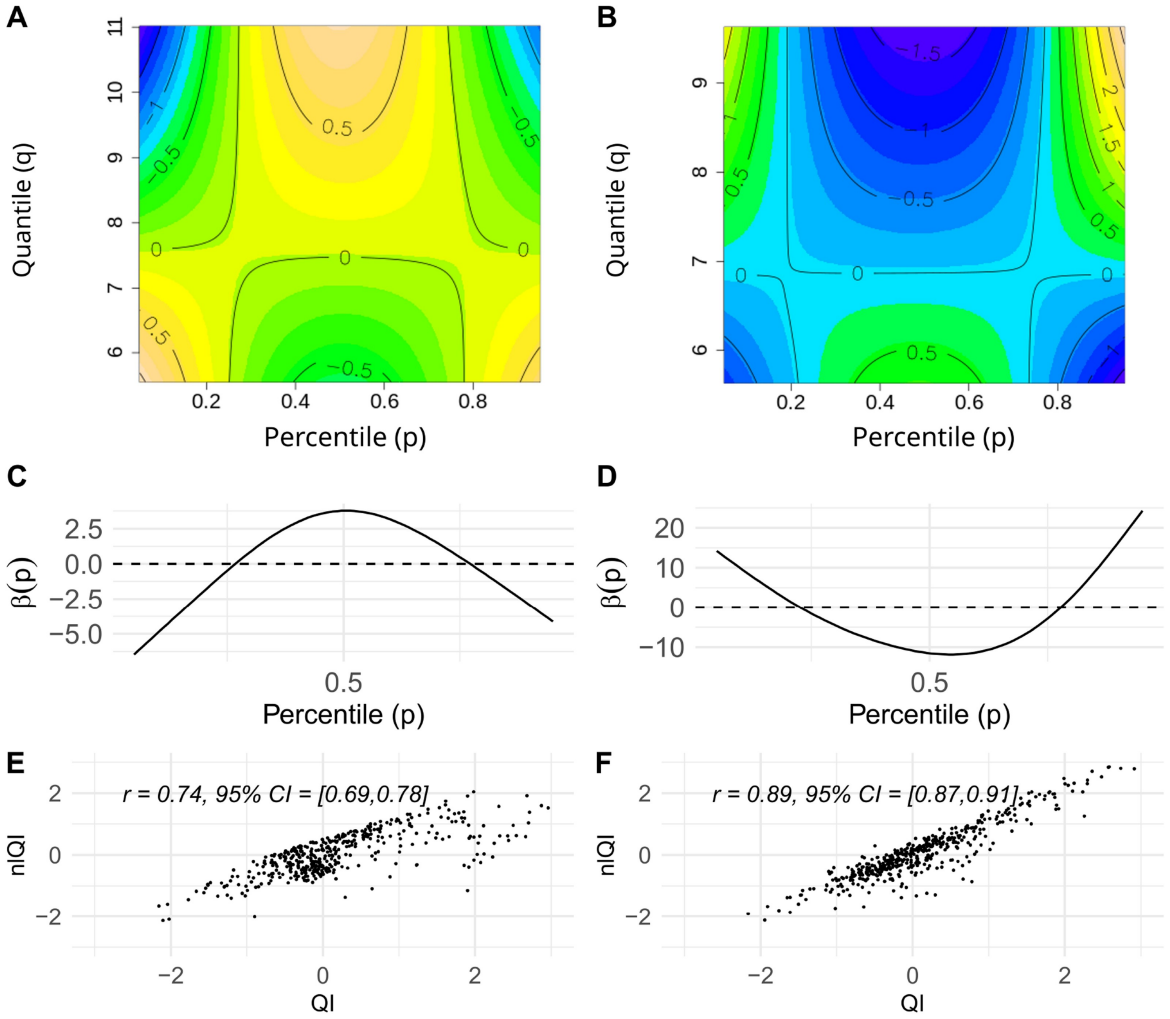
Contour plots of fitted AFCM surfaces for ER (A) and TS (B), weighting functions estimated from LFCM models for ER (C) and TS (D), and scatter plots of *nlQI* versus *QI* for ER (E) and TS (F).

Figure 1A and B presents the contour plots of the estimated F(p,q) surface obtained by fitting AFCM models to the training dataset. For ER, the contour plot shows that the highest levels of surface $\hat{F(p,q)}$ correspond to the middle percentiles (0.4<p<0.6) and high values of the corresponding quantiles (9<q<11). Thus, the quantile functions with higher medians and other middle percentiles yield higher values of nlQIs. Figure 1C shows the estimated weighting function $\hat{\beta (p)}$ for ER *QIs* based on the LFCM model. Similar to $\hat{F(p,q)}$ surface, $\hat{\beta (p)}$ gives the highest weights to middle percentiles (0.4<p<0.6), but the corresponding quantiles can have any values, not necessarily on the higher end. For TS, the contour plot in Fig. 1B shows that the highest levels of $\hat{F(p,q)}$ correspond to the upper tail percentiles (p>0.8) with the highest values of the corresponding quantiles. Thus, the quantile functions with higher upper tails of the CSI distributions yield higher values of nlQIs. Figure 1D shows $\hat{\beta (p)}$ for TS *QIs* based on the LFCM model. Again, consistently with the $\hat{F(p,q)}$ surface, $\hat{\beta (p)}$ gives the highest weights to the upper tail percentiles (p>0.8), but not necessarily with the highest values of corresponding quantiles. The *QIs* and nlQIs are positively associated for both ER and TS (Fig. 1E and F).

Table 1 presents the results of univariable or multivariable Cox models fitted to the test set with each considered biomarker as a predictor of PFS. For ER, the effect size [hazard ratio (HR)] was very similar and significant in univariate models for quantile-based and spatial biomarkers, but not significant for ER MSI. The HRs were reduced and nonsignificant in multivariable models adjusted for tumor size and histological grade. For TS, only *nlQI* and *QI* biomarkers were significant predictors of PFS univariately, and only *nlQI* remained significant in the multivariable model. Notably, the spatial markers yielded just slightly higher effect sizes than QI biomarkers for ER, for which the null hypothesis of independence between CSIs and cell locations was rejected for 82% of the tissues. Meanwhile, for TS CSIs, the null hypothesis of independence from cell locations was rejected only for 64% of the tissues, and the effect sizes were higher for the proposed *nlQI* and *QI* than for the spatial markers.

**Table 1.** Protein biomarkers as predictors of PFS in univariate and multivariable Cox models fitted to the test set data.^a^

	Univariable				Multivariable			
	*nlQI*				*nlQI*			
	HR	95% HR CI		*P*-value	HR	95% HR CI		*P*-value
ER	0.65	0.44	0.98	.038	0.80	0.52	1.23	.308
TS	1.31	1.07	1.61	.009	1.23	0.99	1.52	.063

	QI				QI			
	HR	95% HR CI		*P*-value	HR	95% HR CI		*P*-value
ER	0.70	0.50	0.99	.040	0.80	0.57	1.14	.216
TS	1.24	1.04	1.47	.014	1.13	0.94	1.37	.203

	MSI				MSI			
	HR	95% HR CI		*P*-value	HR	95% HR CI		*P*-value
ER	0.78	0.59	1.03	.083	0.85	0.65	1.11	.224
TS	1.00	0.79	1.27	.996	0.95	0.78	1.16	.623

	*AUcMean*				*AUcMean*			
	HR	95% HR CI		*P*-value	HR	95% HR CI		*P*-value
ER	0.60	0.40	0.91	.015	0.76	0.50	1.16	.204
TS	1.06	0.831	1.36	.633	1.03	0.83	1.29	.774

	*AUcVar*				*AUcVar*			
	HR	95% HR CI		*P*-value	HR	95% HR CI		*P*-value
ER	0.62	0.40	0.94	.024	0.73	0.48	1.09	.126
TS	1.01	0.97	1.05	.622	1.01	0.95	1.06	.853

^a ^Multivariable models also include tumor size and histological grade.

### 3.2 Prediction of high mitotic index

For dichotomized high (score 3) vs low (scores 1–2) of pathologist’s evaluated mitotic index, Ki67, and CyclinD3 CSI quantile functions were significant as functional predictors in the FGAM model. Ki67 is a protein expressed during cell division (G1 phase through M phase) and is commonly used as a cell proliferation marker, as it is absent in nondividing cells. CyclinD3 is a protein expressed during the transition from the G1 phase to the S phase, activating cyclin-dependent kinases (CDK4 and CDK6) and driving cell cycle progression. We have previously reported the results for Ki67 CSI quantile functions as predictors of PFS (Yi *et al.* 2023a) but not mitotic index.

The null hypothesis of independence between CSIs and locations of cells was rejected for 72% of ER images and 63% of CyclinD3 images.

**Figure 2.**
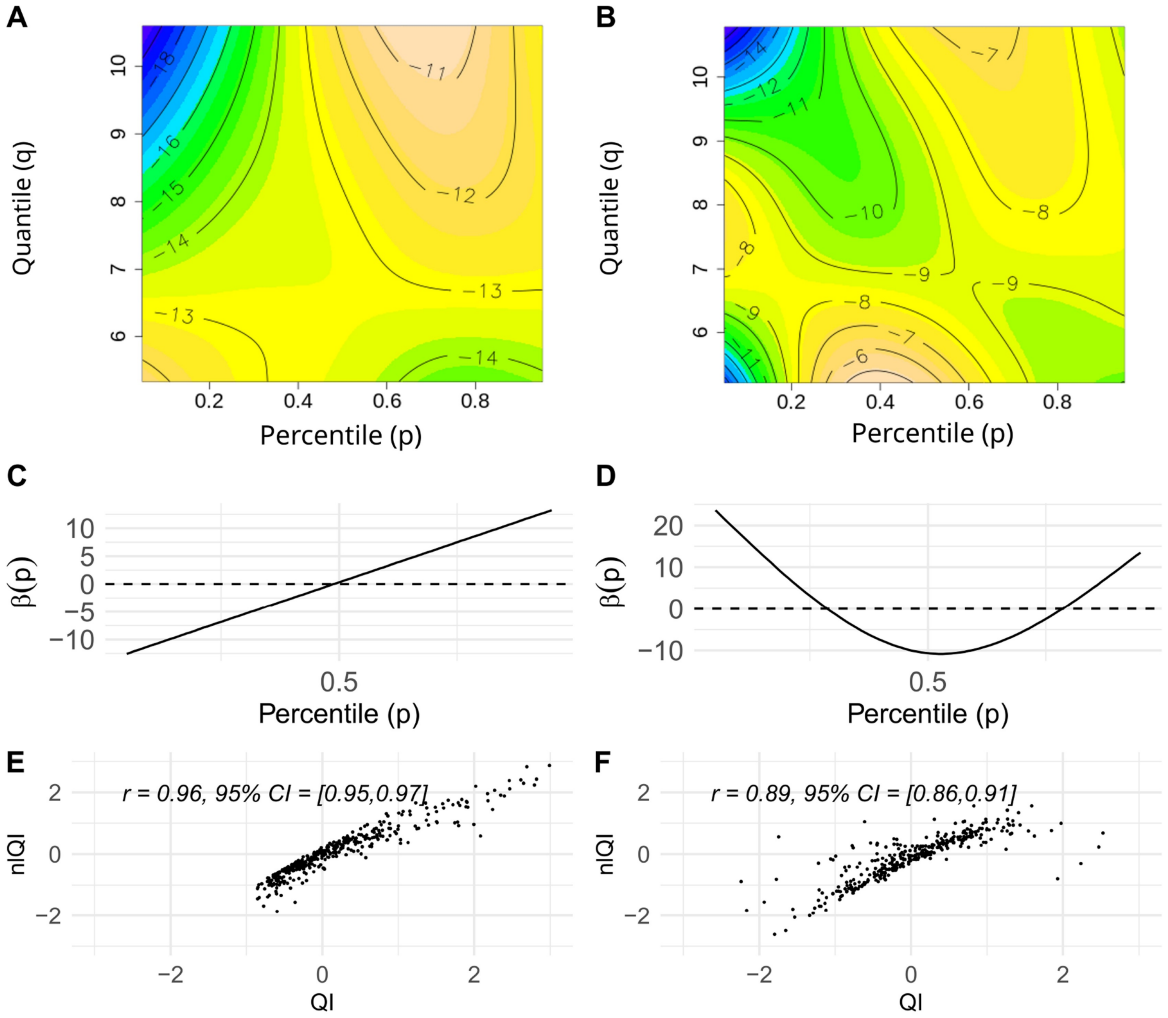
Contour plots of FGAM surfaces for Ki67 (A) and CyclinD3 (B), FGLM-estimated weighting functions for Ki67 (C) and CyclinD3 (D), scatter plots of *nlQI* versus *QI* for Ki67 (E) and CyclinD3 (F).

Figure 2A and B presents the contour plots of estimated F(p,q) surface obtained by fitting FGAM models to a training dataset. For Ki67, the contour plot shows the highest $\hat{F(p,q)}$ surface values for the points corresponding to upper percentiles (p>0.5) and high values of the corresponding quantiles.

Figure 2C shows the weighting function $\hat{\beta (p)}$ for Ki67 *QIs* based on the FGLM model. Similar to $\hat{F(p,q)}$ surface, $\hat{\beta (p)}$ gives the highest weights to the fourth quartile (p>0.75), but the corresponding quantiles can have any values, not necessarily on the higher end. Respectively, CSI distributions with wider ranges and higher levels of Ki67 in the upper quantiles yield the highest *QIs*.

For CyclinD3, the contour plot (Fig. 2B) shows the highest $\hat{F(p,q)}$ values for the points corresponding to upper percentiles (p>0.5) and high values of the corresponding quantiles, as well as for the points corresponding to middle percentiles (0.2<p<0.6) with low values of the corresponding quantiles. Thus, the quantile functions with higher CSI values in quantiles above the median odds ratio (OR) with low CSI values in quantiles with middle percentiles (0.2<p<0.6) yield higher values of nlQIs. Figure 2D shows the weighting function $\hat{\beta (p)}$ for CyclinD3 *QIs* based on the FGLM model.

In contrast to $\hat{F(p,q)}$ surface, $\hat{\beta (p)}$ gives negative weight to the quantiles corresponding to middle percentiles (0.3<p<0.7).

Table 2 presents the results of univariable or multivariable logistic regression models fitted to the test set with each considered biomarker as a predictor of high mitotic index. All Ki67 biomarkers were significant predictors of high mitotic index in univariable or multivariable models, but the *nlQI* biomarker yielded the highest effect size (OR), followed by the effect sizes for AUcMean and *QI* biomarkers. For CyclinD3, only *QI* and *nlQI* biomarkers were significant predictors of high mitotic index, and the *nlQI* biomarker yielded a higher effect size than the *QI* biomarker in univariable and multivariable models.

**Table 2.** Protein biomarkers of high mitotic index in univariate and multivariable logistic regression model fitted to the test set data.^a^

	Univariate				Multivariable			
	*nlQI*				*nlQI*			
	OR	95% OR CI		*P*-value	OR	95% OR CI		*P*-value
Ki67	3.20	2.28	4.68	< .001	2.93	2.05	4.32	< .001
CyclinD3	2.15	1.46	3.28	< .001	2.24	1.45	3.58	< .001

	QI				QI			
	OR	95% OR CI		*P*-value	OR	95% OR CI		*P*-value
Ki67	2.30	1.76	3.09	< .001	2.15	1.62	2.91	< .001
CyclinD3	1.33	0.95	1.89	.096	1.53	1.04	2.27	.032

	MSI				MSI			
	OR	95% OR CI		*P*-value	OR	95% OR CI		*P*-value
Ki67	1.34	1.14	1.60	.001	1.27	1.08	1.53	.009
CyclinD3	1.05	0.94	1.17	.405	1.09	0.96	1.23	.157

	AUcMean				AUcMean			
	OR	95% OR CI		*P*-value	OR	95% OR CI		*P*-value
Ki67	2.56	1.87	3.59	< .001	2.41	1.68	3.54	< .001
CyclinD3	1.02	0.77	1.34	.876	1.23	0.89	1.69	.212

	AUcVar				AUcVar			
	OR	95% OR CI		*P*-value	OR	95% OR CI		*P*-value
Ki67	1.49	1.22	1.84	< .001	1.44	1.13	1.87	.004
CyclinD3	0.92	0.68	1.23	.596	0.97	0.69	1.38	.882

a Multivariable models also include hormone receptor status and tumor size.

### 3.3 Simulation study

**Figure 3.**
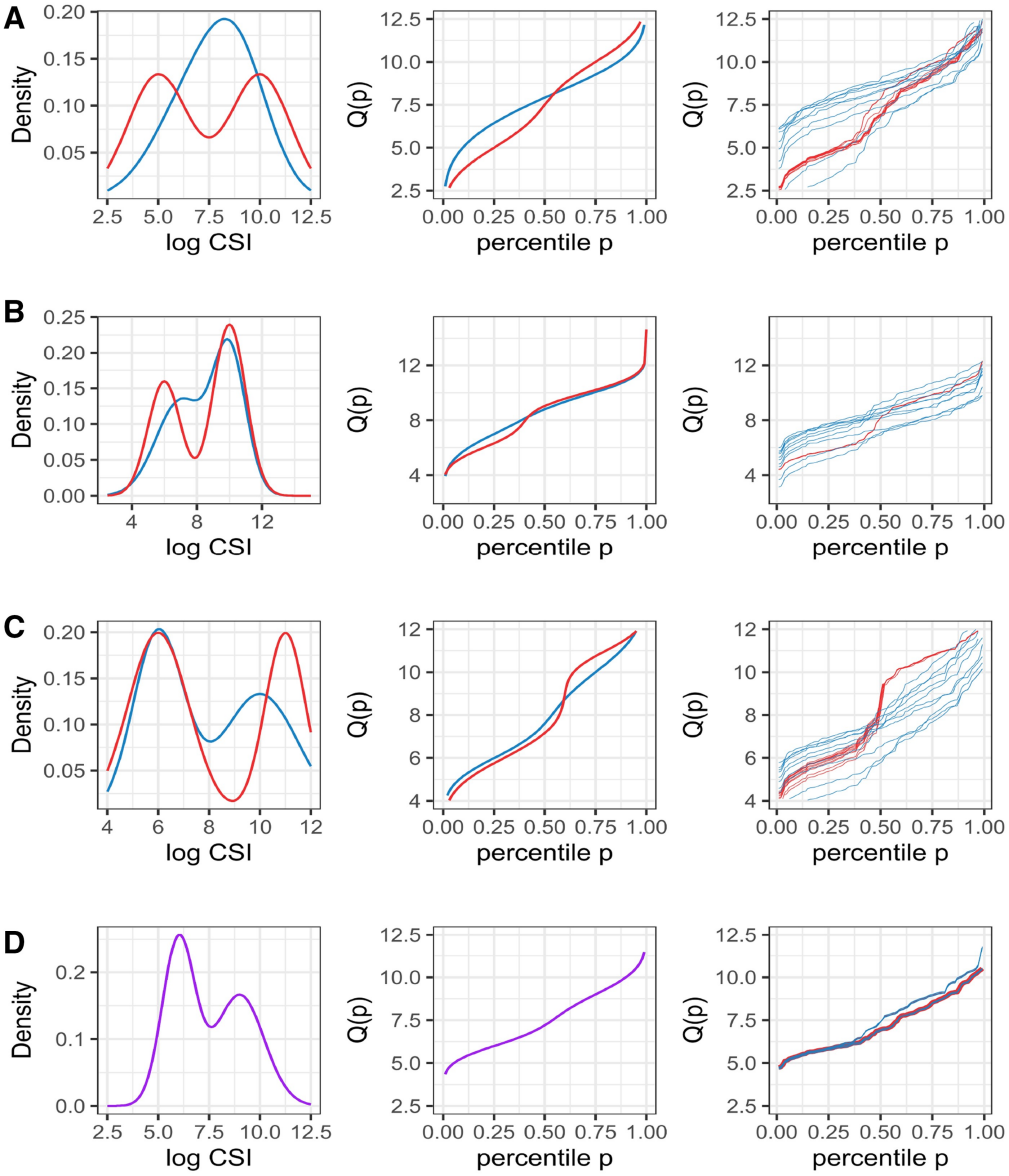
Average density, quantile functions, and representative sample empirical quantile functions for the high-risk (red) and low-risk (blue) groups for scenarios A, B, and C in, respectively, labeled subplots. For scenario D, the high-risk and low-risk groups had the same average density and quantile functions of CSI distributions (purple lines).

For each subject, we simulated 20, 60, 100, or 200 CSI observations per group from 2-component mixtures of normal distributions with random subject effects in all mixture parameters. Different patterns of average quantile functions and random subject effects were associated with groups designated as high risk versus low risk as detailed in Supplementary Table S1. Figure 3 shows density plots and corresponding average quantile functions and representative sample empirical quantile functions for high-risk and low-risk groups used in four simulated scenarios that exemplify CSI distributions observed in real mIF-IHC data. In scenario A, the high-risk group has a distinctive bi-modality, while the two mixture components of the low-risk groups form a unimodal distribution. This scenario mimics the situation when the high-risk group has a distinctive component of high protein expression levels. In scenario B, high-risk and low-risk groups have different group parameters of the first mixture component, but similar parameters of the second component. This scenario mimics the situation when the loss of protein expression is associated with high risk. In scenario C, high-risk and low-risk groups have different group parameters of the second mixture component, but similar parameters of the first component. This scenario mimics the situation when some proportion of higher protein expression levels is associated with the high risk. Scenario D assumed no difference in CSI distributions between high-risk and low-risk groups.

Binary outcomes were simulated using the binomial distribution with probability of event 0.61 for the high-risk group, with the corresponding OR of 2.5. Survival outcome data were simulated using simsurv R package under the assumption of Weibull distribution with parameters specified in the Supplementary Table S2. For each scenario, we generated 200 pairs of training and test datasets with 40–240 subjects per group. In each pair, the training set was used to estimate the weighting function β(p) and surface F(p,q). These estimates were used to compute *QIs* and *nlQIs* for all subjects in the test set. The quantile function for each subject was tabulated (i) using 19 quantiles corresponding to percentiles 5%–95% with a step 5% or (ii) using 99 quantiles from 1% to 99% with a step 1%. In addition to *QIs* and *nlQIs*, we also computed the MSI and the OQ biomarker (Yi *et al.* 2023b). Performance of the biomarkers was evaluated by computing the area under the receiver operating characteristic curve (AUC-ROC) (Fawcett 2006) for predicting the high-risk group membership using the R package pROC (Robin *et al.* 2011).

**Figure 4.**
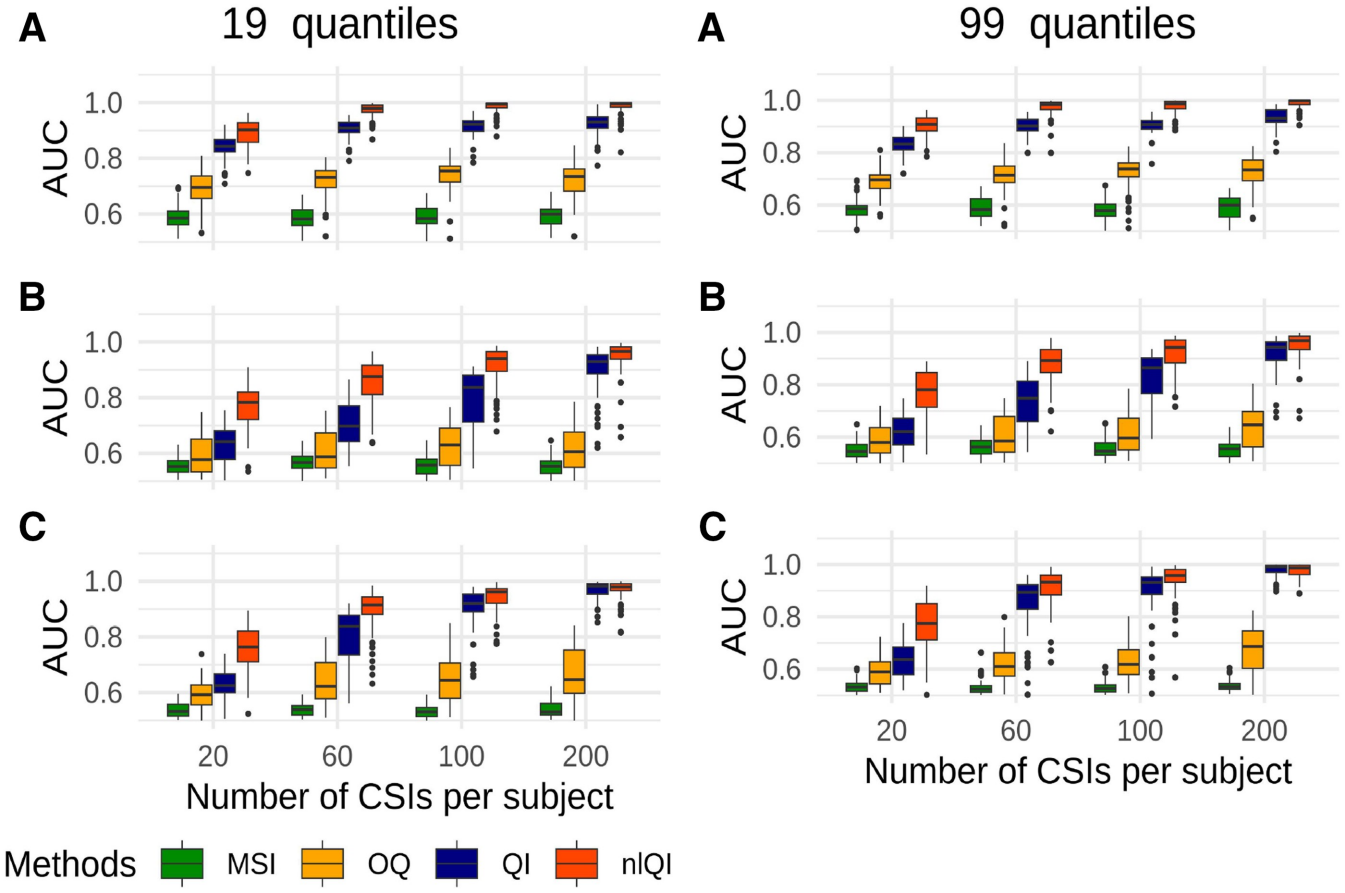
AUC-ROC distributions for biomarkers of the high-risk group trained with survival outcome in simulations under scenarios A, B, and C with 120 subjects per group.

**Figure 5.**
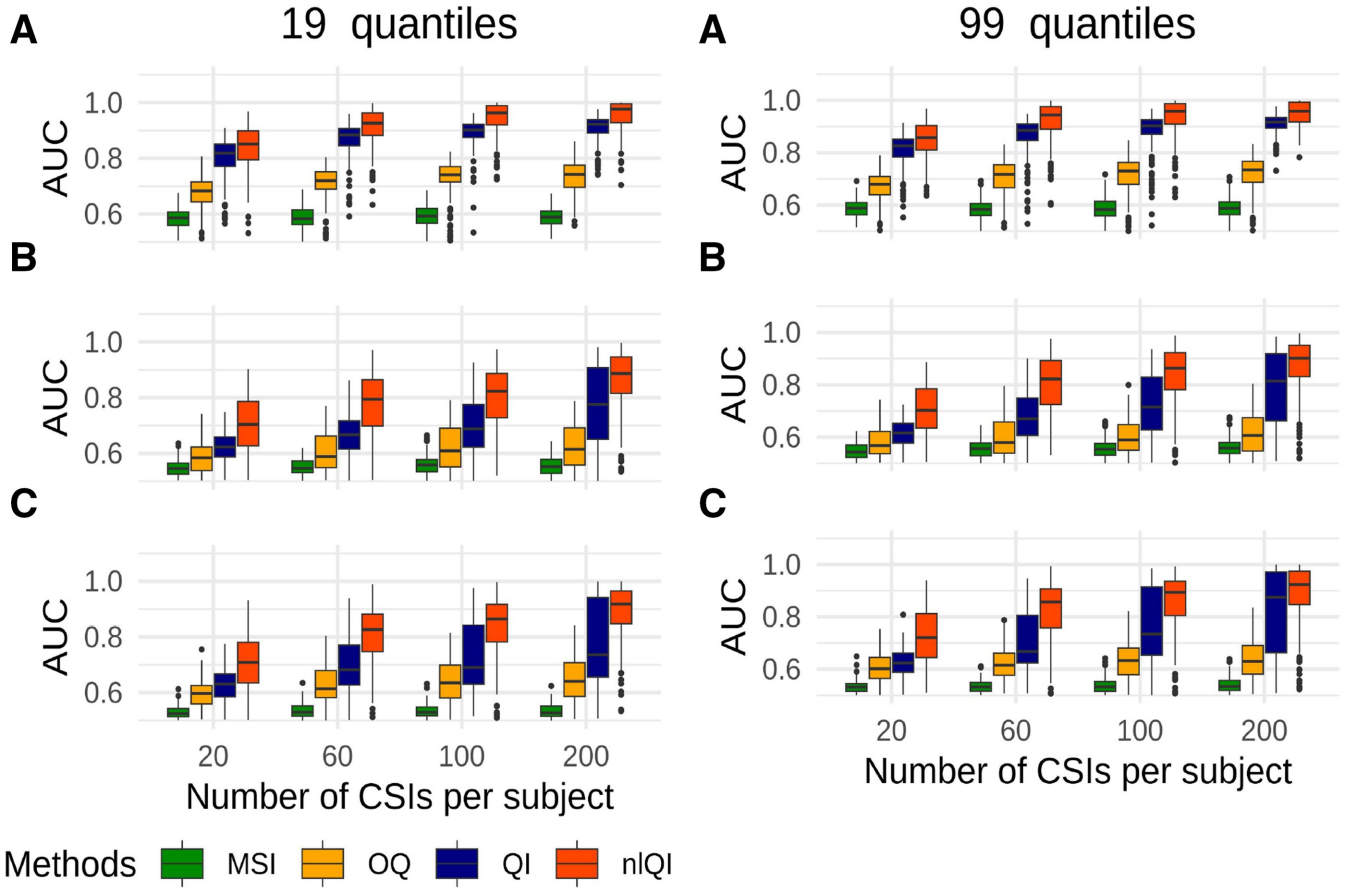
AUC-ROC distributions for biomarkers of the high-risk group trained with binary outcome in simulations under scenarios A, B, and C with 120 subjects per group.

Figure 4 presents the AUC-ROC distributions for biomarkers trained with survival outcome in simulations with 120 subjects per group based on 19 quantiles (left column) or 99 quantiles (right column). Distributions of AUC-ROCs for biomarkers trained with a binary outcome are shown in Fig. 5. Results for all scenarios with 40, 80, 120, or 240 subjects per group, 19 or 99 quantiles, survival or binary outcome are shown in Supplementary Figs S4–S7. Results for Scenario D with no difference in CSI distributions between the high-risk and low-risk groups and no difference in biomarker performance are shown only in the Supplementary Tables. For scenarios A–C, the *nlQI* biomarkers performed better than *QI* biomarkers across all considered numbers of subjects per group and numbers of observations per subject and for both types of outcomes. Generally, larger differences between AUCs of *nlQI* and AUCs of *QI* were observed for scenarios A–C with survival or binary outcome. These differences were higher for simulations with 80–240 subjects per group. Lower differences were observed with 40 subjects per group, likely attributed to the smaller number of parameters in the models used for computing *QIs* as compared to models used for computing *nlQI*. For scenario C with survival outcome, 120–240 subjects per group and 200 CSIs per subject, *nlQI* and *QI* biomarkers yielded similar AUCs close to 1. Both *nlQI* and *QI* biomarkers performed substantially better than MSI and OQ biomarkers for scenarios A–C and across all considered numbers of subjects per group and numbers of observations per subject and for both types of outcomes, but even higher differences for survival outcome. Comparing results based on 99 quantiles versus 19 quantiles, we observe generally negligible differences across all scenarios and sample sizes. Since using 99 quantiles adds computational burden, we recommend using QI biomarkers based on a moderate number of quantiles (19–25).

## 4 Discussion

In this article, we propose novel nonlinear QI biomarkers that capture complex associations between single-cell quantile functions of functional proteins and clinical outcomes of interest. Scalar-on-function regression models offer a theoretical framework for the proposed QI biomarkers, but they are challenging to interpret directly. Our approach provides the means of deriving a single scalar index by fitting a scalar-on-function regression model with a quantile function predictor to a training set. Using the fitted surface of the scalar-on-function regression model, nonlinear quantile indices can be computed for subjects in a test dataset and used in the same way as familiar continuous scalar predictors.

The main limitation of the proposed approach is the need to split data into the training and test sets. TMA data usually include a large number of independent tissues, but mIF-IHC studies with whole tissue samples tend to have smaller numbers of independent tissues with multiple regions of interest (ROIs) per tissue. Also, for experiments with multiple batches of whole tissue slides, there is a need to normalize CSI data as described in Harris *et al.* (2022) before computing the QI biomarkers to eliminate possible confounding between experimental processing and biological differences.

For computing *QI* and *nlQI* biomarkers, we used quantiles corresponding to equally spaced percentiles. Trimming a higher percentage from the distribution tails will make *QIs* and *nlQIs* more robust to potential outliers. With multiple ROIs per tissue, ROI-specific quantile functions should be aggregated into one function per tissue to be used for computing the QI biomarker. We have implemented computation of point-wise minimum, median, mean and maximum for aggregating ROI-specific quantile functions. We have also implemented a cross-fitting algorithm with splitting one dataset into k folds and computing *nlQIs* or *QIs* in each fold based on the model fitted to all other folds combined.

Spatial analysis of single-cell mIF-IHC data is becoming standard for studies with *phenotypic markers* defining various subpopulations of cells. Meanwhile, for *functional protein markers*, the choice is not obvious between the simplest MSI, nonspatial analysis of single-cell CSI distributions, and methods that account for the spatial localization of cells. Formal testing of independence between CSI values and spatial locations tends to yield mixed results, with variable proportions of tissues with rejected hypothesis of independence, depending on the protein. As spatial approaches for analysis of functional protein markers are still limited, complex, and computationally intensive, nonspatial alternatives based on distributions of functional protein CSI levels may outperform spatial approaches. For some functional proteins, optimal utilization of actual expression levels may be more important than accounting for spatial heterogeneity. Our results provide empirical evidence that the proposed nlQIs biomarkers may yield better prognostic results than spatial biomarkers if the hypothesis of independence between CSI and cell locations is rejected in less than 80% of the tissues. However, ignoring spatial information may not provide optimal functional protein biomarkers when there is strong evidence of dependence between CSI and cell locations in majority of the tissues.

In this work, the proposed methodology was used for the analysis of functional proteins in cancer cells, but the same approach can be applied to derive QI biomarkers based on protein expression in other cell phenotypes. Our methodology is suitable for any single-cell expressions of proteins or mRNAs, including flow cytometry or scRNA sequencing data.

## Supplementary Material

btaf182_Supplementary_Data

## Data Availability

The biomarker data analyzed are available in R package Qindex at https://CRAN.R-project.org/package=Qindex.
