# Supplementary material for: Functional protein biomarkers based on distributions of expression levels in single-cell imaging data
Source: Bioinformatics. 2025 Apr 21;41(5):btaf182. doi: 10.1093/bioinformatics/btaf182 (PMC12070390; doi:10.1093/bioinformatics/btaf182)
Supplement: btaf182_Supplementary_Data [file btaf182_supplementary_data.pdf]

Supplementary Material

1 Real data

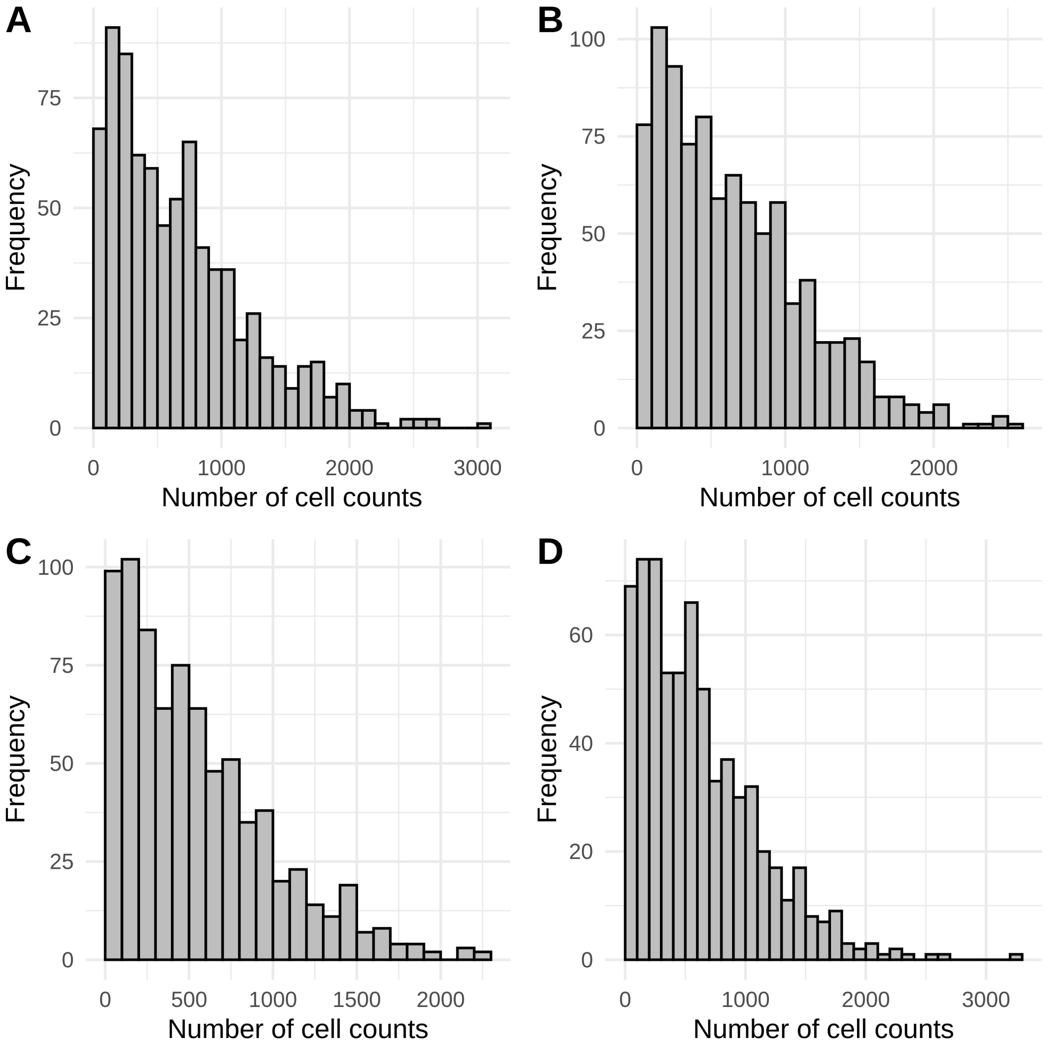

Figure S1: Histogram of cell counts for ER (A), TS (B), Ki67 (C), and CyclinD3 (D)

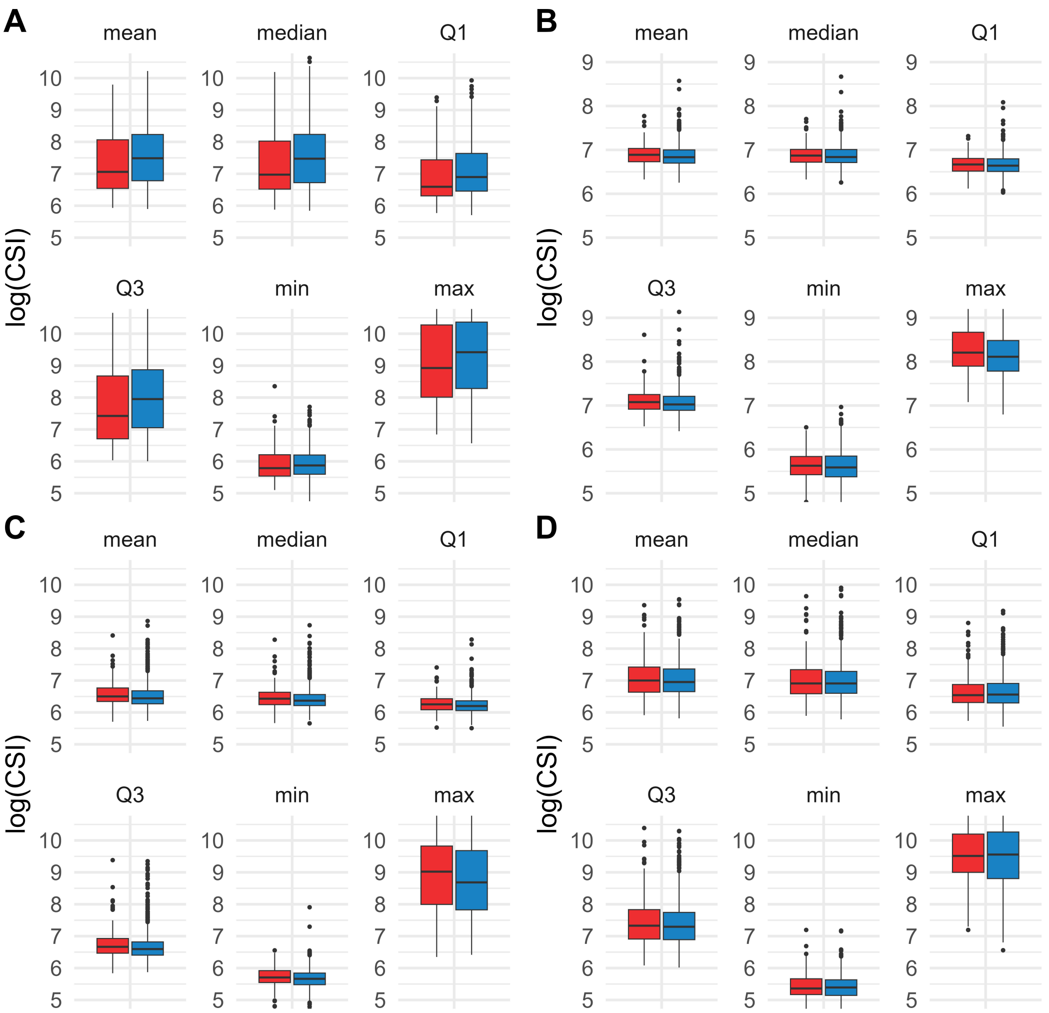

Figure S2: Box plots of mean, median, the first and third quartiles, minimum, and maximum of log(CSI) by recurrence (recurrence: red, no recurrence: blue) for ER (A) and TS (B) and those by mitotic index status (high mitotic index: red, low mitotic index: blue)for Ki67 (C) and CyclinD3 (D)

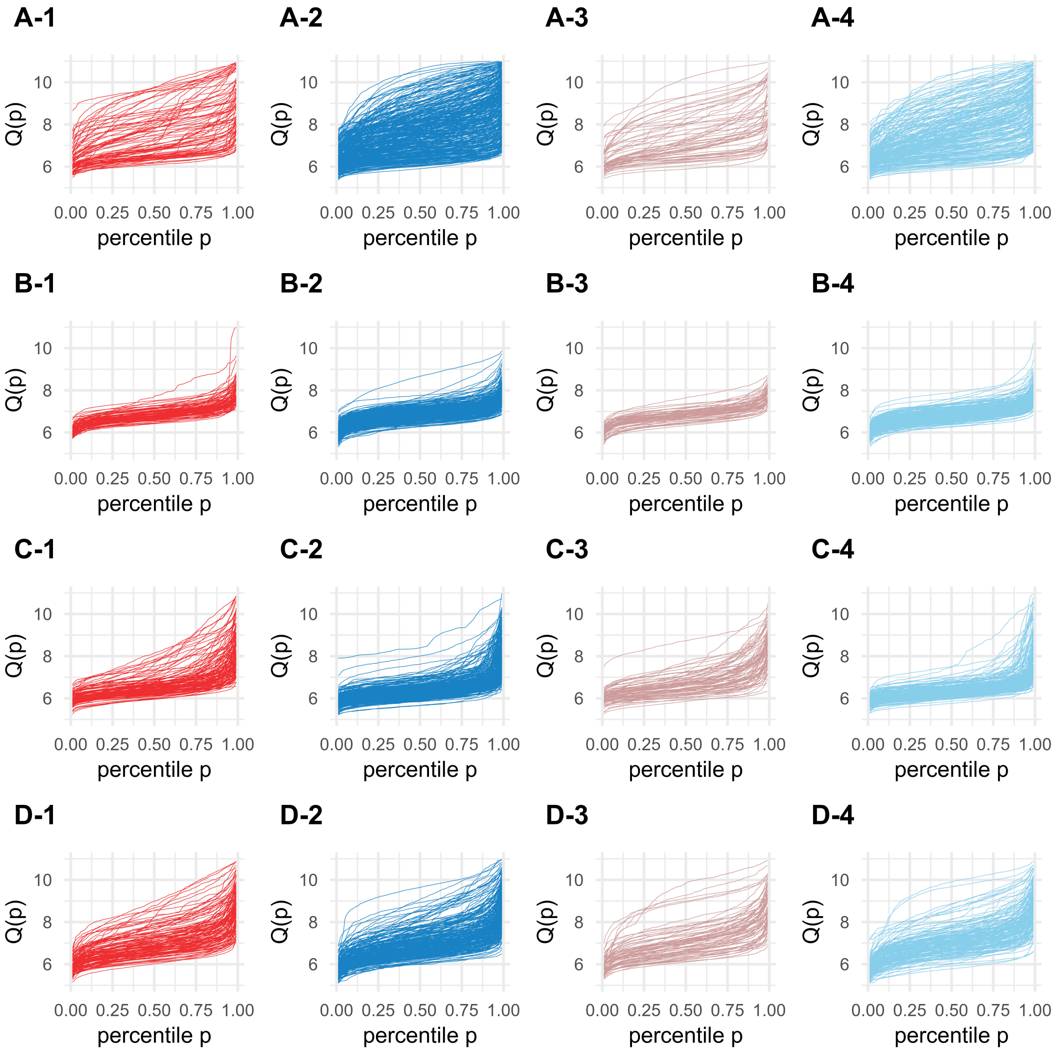

Figure S3: Empirical quantile functions for subjects experienced recurrence in training set (red), no recurrence in training set (blue), recurrence in test set (pink), and no recurrence in test set (sky blue) for ER (A-1, A-2, A-3, A-4) and for TS (B-1, B-2, B-3, B-4). Empirical quantile functions for subjects who had high mitotic index in training set (red), low mitotic index in training set (blue), high mitotic index in test set (pink), and low mitotic index in test set for Ki67 (C-1, C-2, C-3, C-4) and for CyclinD3 (D-1, D-2, D-3, D-4)

2 Simulation study

2.1 Simulation design

Scenario	Risk	Parameters				
		**A1**	**B1**	**w2**	**A2**	**B2**
[A] Difference between high and low risk in the LOWER and UPPER tail	Low	7	2.0	0.4	9	1.5
	High	5	1.5	0.5	10	1.5
[B] Difference between high and low risk in the LOWER tail	Low	7	1.5	0.5	10	1
	High	6	1	0.6	10	1
[C] Difference between high and low risk in the UPPER tail	Low	6	1	0.5	10	1.5
	High	6	1.2	0.4	11	0.8
[D] NO difference between high and low risk	Low	6	0.8	0.5	9	1.2
	High	6	0.8	0.5	9	1.2

Table S1: Parameters of Normal mixtures to generate cell signal intensities used in each simulated scenario

Scenario A low risk group: For ith subject, $A1_i = A1 + \mu_i$ where $\mu_i \sim N(0, 1.5^2)$, $B1_i = B1 + \sigma_i$ where $\sigma_i \sim N(0, 0.01^2)$, $A2_i = A2 + \nu_i$ where $\nu_i \sim N(0, 0.5^2)$, $B2_i = B2 + \tau_i$ where $\tau_i \sim N(0, 0.01^2)$, and $w1_i = (1 - w2_i) + p_i$ where $p_i \sim N(0, 0.1^2)$
Scenario A high risk group: For ith subject, $A1_i = A1 + \mu_i$ where $\mu_i \sim N(0, 0.3^2)$, $B1_i = B1 + \sigma_i$ where $\sigma_i \sim N(0, 0.01^2)$, $A2_i = A2 + \nu_i$ where $\nu_i \sim N(0, 0.3^2)$, $B2_i = B2 + \tau_i$ where $\tau_i \sim N(0, 0.01^2)$, and $w1_i = (1 - w2_i) + p_i$ where $p_i \sim N(0, 0.1^2)$
Scenario B low risk group: For ith subject, $A1_i = A1 + \mu_i$ where $\mu_i \sim N(0, 1^2)$, $B1_i = B1 + \sigma_i$ where $\sigma_i \sim N(0, 0.01^2)$, $A2_i = A2 + \nu_i$ where $\nu_i \sim N(0, 1^2)$, $B2_i = B2 + \tau_i$ where $\tau_i \sim N(0, 0.01^2)$, and $w1_i = (1 - w2_i) + p_i$ where $p_i \sim N(0, 0.1^2)$
Scenario B high risk group: For ith subject, $A1_i = A1 + \mu_i$ where $\mu_i \sim N(0, 0.1^2)$, $B1_i = B1 + \sigma_i$ where $\sigma_i \sim N(0, 0.01^2)$, $A2_i = A2 + \nu_i$ where $\nu_i \sim N(0, 0.1^2)$, $B2_i = B2 + \tau_i$ where $\tau_i \sim N(0, 0.01^2)$, and $w1_i = (1 - w2_i) + p_i$ where $p_i \sim N(0, 0.05^2)$
Scenario C low risk group: For ith subject, $A1_i = A1 + \mu_i$ where $\mu_i \sim N(0, 1^2)$, $B1_i = B1 + \sigma_i$ where $\sigma_i \sim N(0, 0.01^2)$, $A2_i = A2 + \nu_i$ where $\nu_i \sim N(0, 1^2)$, $B2_i = B2 + \tau_i$ where $\tau_i \sim N(0, 0.01^2)$, and $w1_i = (1 - w2_i) + p_i$ where $p_i \sim N(0, 0.1^2)$
Scenario C high risk group: For ith subject, $A1_i = A1 + \mu_i$ where $\mu_i \sim N(0, 0.5^2)$, $B1_i = B1 + \sigma_i$ where $\sigma_i \sim N(0, 0.01^2)$, $A2_i = A2 + \nu_i$ where $\nu_i \sim N(0, 0.1^2)$, $B2_i = B2 + \tau_i$ where $\tau_i \sim N(0, 0.01^2)$, and $w1_i = (1 - w2_i) + p_i$ where $p_i \sim N(0, 0.1^2)$
Scenario D low risk group: For ith subject, $A1_i = A1 + \mu_i$ where $\mu_i \sim N(0, 0.2^2)$, $B1_i = B1 + \sigma_i$ where $\sigma_i \sim N(0, 0.05^2)$, $A2_i = A2 + \nu_i$ where $\nu_i \sim N(0, 0.2^2)$, $B2_i = B2 + \tau_i$ where $\tau_i \sim N(0, 0.01^2)$, and $w1_i = (1 - w2_i) + p_i$ where $p_i \sim N(0, 0.01^2)$
Scenario D high risk group: For ith subject, $A1_i = A1 + \mu_i$ where $\mu_i \sim N(0, 0.2^2)$, $B1_i = B1 + \sigma_i$ where $\sigma_i \sim N(0, 0.05^2)$, $A2_i = A2 + \nu_i$ where $\nu_i \sim N(0, 0.2^2)$, $B2_i = B2 + \tau_i$ where $\tau_i \sim N(0, 0.01^2)$, and $w1_i = (1 - w2_i) + p_i$ where $p_i \sim N(0, 0.01^2)$

**Risk group**	**Event indicator**	**Weibull distribution parameters**	
		**Shape**	**Scale**
Low	Event	5.1	2
	Censored	5	1
High	Event	4.5	2
	Censored	5	1

Table S2: Parameters of Weibull distribution to generate event time in survival analysis

2.2 Simulation results for survival outcomes

Description of scenarios A-D is written in Supplementary Table 2.1.

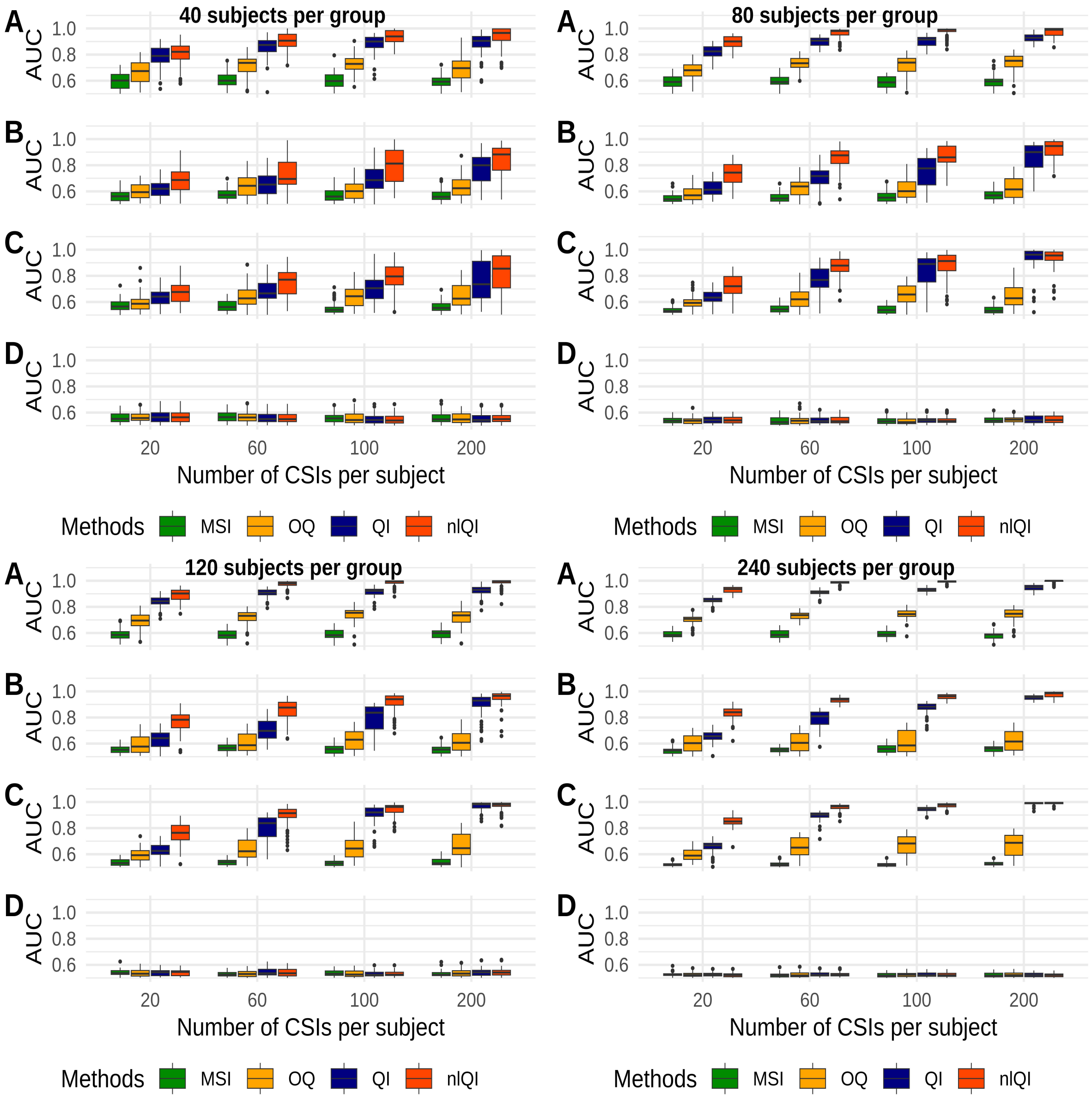

Figure S4: For survival outcome, distribution of AUC-ROCs over 200 simulations by scenario and the number of CSI per subject (19 quantiles).

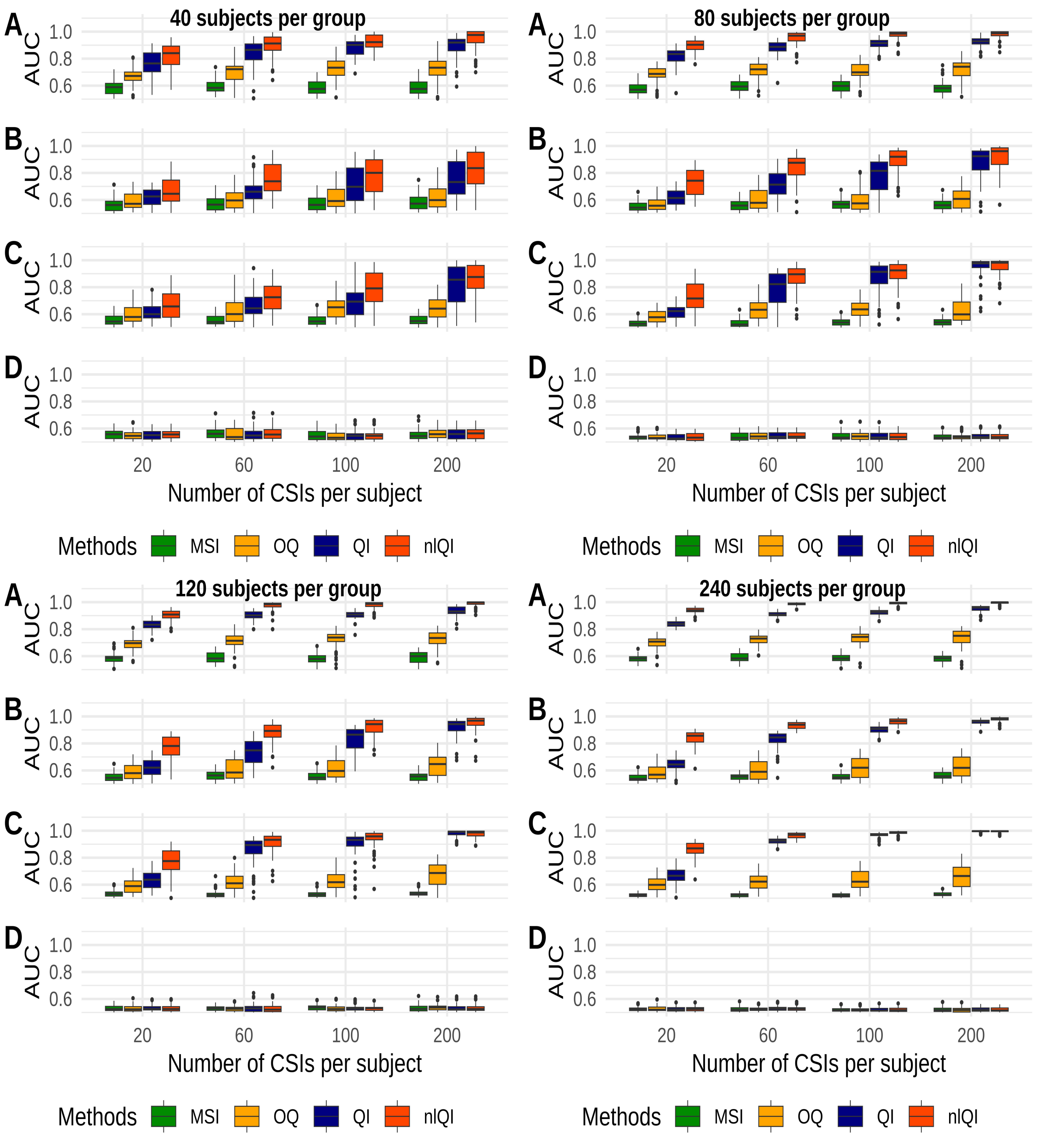

Figure S5: For survival outcome, distribution of AUC-ROCs over 200 simulations by scenario and the number of CSI per subject (99 quantiles).

2.2.1 Simulation results for binary outcomes

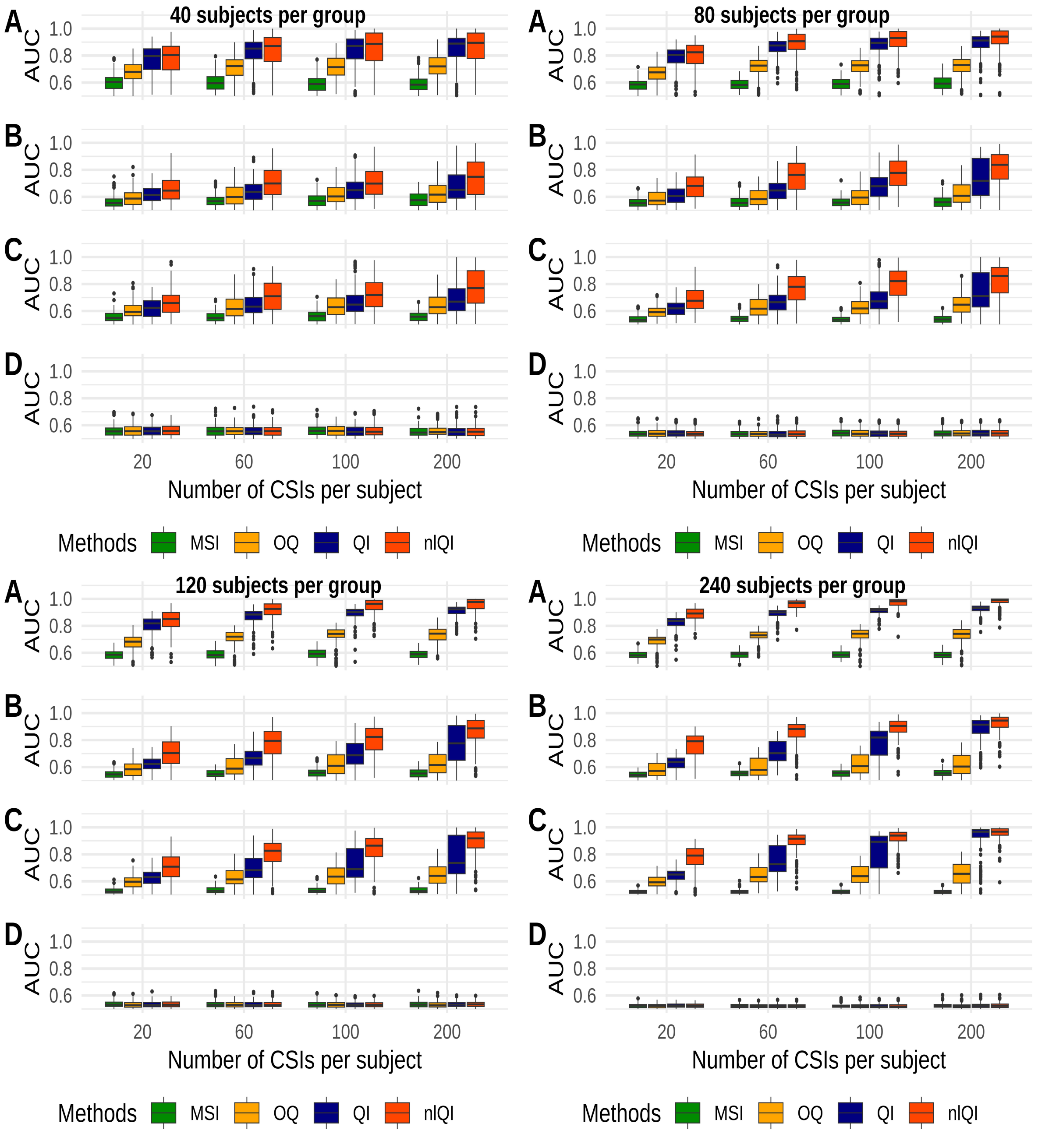

Figure S6: For binary outcome, distribution of AUC-ROCs over 200 simulations by scenario and the number of CSI per subject (19 quantiles).

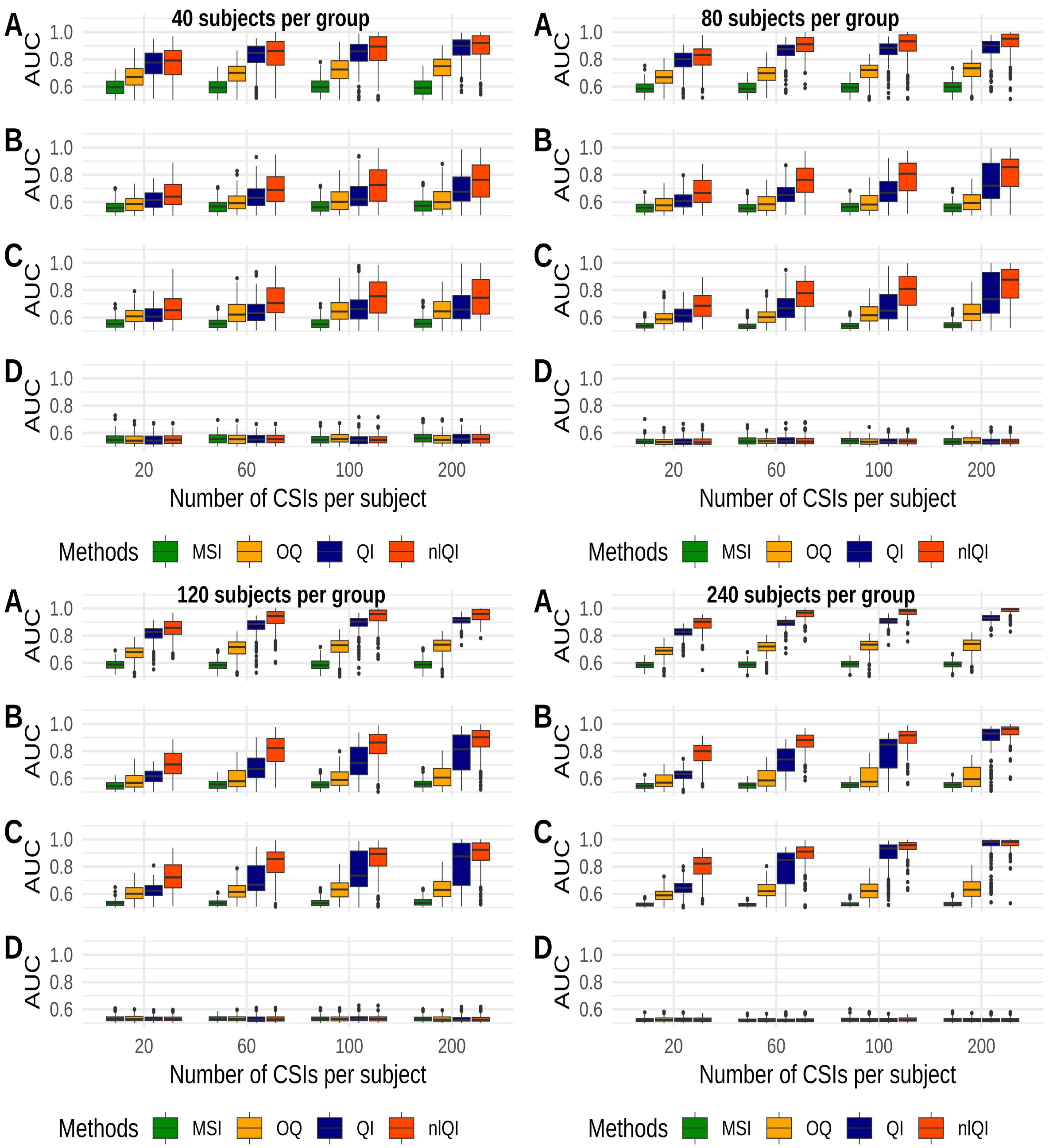

Figure S7: For binary outcome, distribution of AUC-ROCs over 200 simulations by scenario and the number of CSI per subject (99 quantiles).
